# A malware detection system using a hybrid approach of multi-heads attention-based control flow traces and image visualization

**DOI:** 10.1186/s13677-022-00349-8

**Published:** 2022-11-03

**Authors:** Farhan Ullah, Gautam Srivastava, Shamsher Ullah

**Affiliations:** 1 School of Software, Northwestern Polytechnical University, Xian, 710072 China; 2 Department of Math and Computer Science, Brandon University, R7A 6A9 Brandon, Canada; 3 Research Centre for Interneural Computing, China Medical University, 40402 Taichung, Taiwan; 4 Department of Computer Science and Math, Lebanese American University, 1102 Beirut, Lebanon

**Keywords:** Android malware, Control flow graph, Malware visualization, Transfer learning, Ensemble learning, Cybersecurity

## Abstract

Android is the most widely used mobile platform, making it a prime target for malicious attacks. Therefore, it is imperative to effectively circumvent these attacks. Recently, machine learning has been a promising solution for malware detection, which relies on distinguishing features. While machine learning-based malware scanners have a large number of features, adversaries can avoid detection by using feature-related expertise. Therefore, one of the main tasks of the Android security industry is to consistently propose cutting-edge features that can detect suspicious activity. This study presents a novel feature representation approach for malware detection that combines API-Call Graphs (ACGs) with byte-level image representation. First, the reverse engineering procedure is used to obtain the Java programming codes and Dalvik Executable (DEX) file from Android Package Kit (APK). Second, to depict Android apps with high-level features, we develop ACGs by mining API-Calls and API sequences from Control Flow Graph (CFG). The ACGs can act as a digital fingerprint of the actions taken by Android apps. Next, the multi-head attention-based transfer learning method is used to extract trained features vector from ACGs. Third, the DEX file is converted to a malware image, and the texture features are extracted and highlighted using a combination of FAST (Features from Accelerated Segment Test) and BRIEF (Binary Robust Independent Elementary Features). Finally, the ACGs and texture features are combined for effective malware detection and classification. The proposed method uses a customized dataset prepared from the CIC-InvesAndMal2019 dataset and outperforms state-of-the-art methods with 99.27% accuracy.

## Introduction

The prolific growth of the Android platform has encouraged the development of an active developer community. Numerous Android app markets enable the instant download of hundreds of millions of apps. The prevalence of mobile malware threats increases alongside the emergence of Android smartphones and tablets [1]. The Android platform has evolved into the main attack target of malware due to the widespread adoption of the use of Android apps. According to a recent survey [2], the amount of malicious mobile apps and attacks reported in the wild has increased exponentially, posing a significant threat to mobile app markets and users. As a result, there is an urgent need to effectively mitigate them. To combat this challenge, researchers from both industry and academia have developed several methods for detecting malware such as graph-based features using CFG [3, 4], behavior-based [5], signature-based [6], image-based [7], and machine learning approaches [8], etc. Currently, machine learning-based methods are a viable method for malware detection and classification. It can prevent zero-day threats and emerge with malware quickly. However, machine learning-assisted strategies rely heavily on features and classification methods. This study focuses on the effects of features on the effectiveness and performance of malware detection systems.Several methods for extracting features from malware have been developed, including manual and automatic feature engineering techniques. These methods are classified into three categories: static, dynamic, and hybrid. Such features can be used by the models to detect malicious apps and identify their type and class. In addition, it can help to alert users of affected devices about a potential security and privacy breach promptly. Static analysis examines the disassembled script without executing the app to extract syntactic and semantic information by leveraging call graphs [4] and API sequences [3]. Such data can be utilized to generate an archive of signatures needed to recognize common threats and adversarial behaviour patterns. Obfuscation and encryption reduce the effectiveness of static-based techniques by producing multiple variants. Dynamic analysis is based on the concept of observing app activities and behaviour while they are running in virtualization. The mobile OS is a common target for numerous dynamic methods that aim to screen and retrieve personal data. These techniques are effective, but they require a significant amount of computing resources to explore all potential app behaviours [9, 10]. The recent survey [11] on developing features for detecting malicious Android apps thoroughly represented the advantages and shortcomings of cutting-edge techniques. Overall, each feature can only provide a localized view of Android app behaviour and frequently targets a particular type of malware.

In addition, the strength of various features to differentiate between malicious activity varies significantly. As a result, most existing malware detection systems intended to gather a detailed view by utilizing hybrid features rather than a single type. Furthermore, attackers use the existing feature-related experience to develop malware variants to avoid detection. As a result, it is beneficial to build an innovative feature space to supplement existing knowledge and broaden the feature combination space so that attackers have a difficult time evading detection.

### Problem statement

**Fig. 1:**
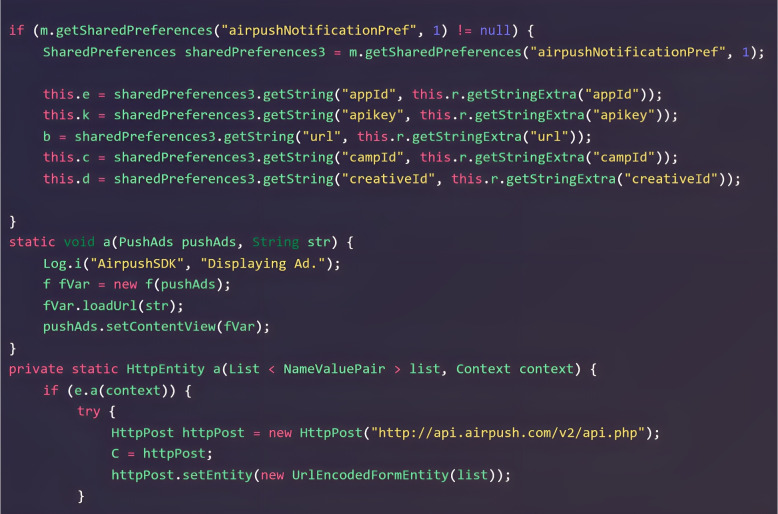
Malicious code snippet in dowgin family of adware

In this research. we combine call-graph and image-based features to develop a novel hybrid feature extraction approach. The Android source code may contain malicious scripts or URLs that compromise the functionalities of the app. CFG-based behavioural segmentation can extract semantic flow from call graphs to target a specific script. Figure 1 depicts a malicious code snippet from the dowgin family for adware. It can be seen that the “airpush” API posts the malicious ads using “static void a (PushAds pushAds, String str)”. Such API can also attempt to obtain detailed information about a genuine app to fully utilize its functions, such as “appId, apikey, url, campId, createId”. Furthermore, the malicious URL^1^ is being used as a host app URL to push the malicious ads. Such semantic patterns cannot be obtained solely via image visualization. Nevertheless, the call-graph evaluation may be affected by code obfuscation, insertion, reshuffling, etc. Image-based malware categorization is extensively employed as it can gather all kinds of structural data, including memory, process, header, etc. Thus, visual images can be utilized to fetch any type of dynamic or obfuscated data. However, it can change the overall hierarchy of an Android file, making it impossible to target a particular malicious code snippet, URL, etc. Aside from that, this technique is entirely based on image characteristics. For instance, hackers can attack the malware images, affecting classification performance. As a result, we combined call-graph features to detect potentially malicious scripts/URLs with textural image features to detect other potentially hazardous tendencies such as memory or resource usage. A hybrid strategy can effectively utilize and classify malicious and benign files.

The main contributions of the paper are the following:

- A reverse engineering method is proposed to prepare a customized dataset. Our custom dataset contains Java sour codes, DEX, and additional resource files.
- The API-Call Graphs (ACGs) features are examined by extraction of CFGs from Java programming code. To extract train features from ACG, the multi-head attention using the Bidirectional Encoder Representations from Transformers (BERT) approach is then proposed.
- The malware-to-image conversion algorithm is intended to convert DEX files to images to analyze the structure of an Android app. The important textural features are extracted and marked using a combination of FAST and BRIEF descriptors.
- The hybrid approach is developed by combining train features extracted from call graphs and textural features for effective malware detection and classification.

The paper is organized as follows: The Section 2 discusses the related work. A detailed explanation of the proposed research work is discussed in the Section 3. The Section 4 explains the experimental implementation of the proposed work with CFG analysis, and multiple comparisons of this research work with different state-of-the-art, and published works. Finally, the Section 5 concludes this paper and discusses further study.

## Literature review

Currently, the Android platform includes several security mechanisms, the most notable of which is the Android permission system, which assists in avoiding malicious programs [12, 13]. Each app must explicitly seek permission from the user during configuration or running time to perform a specific task that requires Android permission, such as location permission. Nevertheless, in the apparent lack of a better knowledge of Android permissions, users frequently grant permission to unidentified apps. As a result, the permission system cannot provide feasible protection. The development of static and dynamic analysis has increased our understanding of malicious behaviours and improved the efficiency and interpretability of the model by reducing the prevalence of arbitrary features. One of the most popular static analysis-based methods for analyzing and classifying malware is to use abstract graph structures such as CFG [14]. It has previously been demonstrated that CFG-based analysis can be combined with machine learning techniques to produce strong malware classification tools [4].

Arslan et al. [15] suggested developing a graphical Android malware detection tool. A one-or-zero vector is extracted from the features of Androidmanifest.xml. To train the CNN network, the feature vector is encoded in two dimensions. These low-level features examine mobile apps in real-time. In terms of detecting malware, the detection rate is 96.2%, with precision at 97.9%, recall at 98.2%, and F-scores at 98.1%. Kumar et al. [16] used bitwise samples for sequentially labelling using the AVClass tool and a clustering method. The malicious program is depicted in grayscale so that local and global textural characteristics can be extracted. The stacking ensemble is then used to classify the malware based on visual features extracted from descriptors. The recommended method has a 98.34% test accuracy. Ma et al. described a technique based on machine learning for detecting Android malware. The features of the CFG are obtained to obtain API details. API calls, frequency, and sequence are used to develop three detection algorithms for Android malware. After that, the graphical features are then fed into the ensemble for malware classification. The detection model has a 98.98% detection accuracy. Frenklach et al. [17] suggested a method for analyzing static Android apps using an app similarity graph (ASG). It is assumed that the key to classifying an app’s activity lies in its generic, reusable major components, such as its functions. The proposed work achieved an accuracy of 97.5% and an AUC score of 98.7% in balanced settings using the Drebin benchmark dataset and a dataset supplied by VirusTotal. Fan et al. [4] used static sensitive sub-graph features for Android malware classification. The proposed method extended a function call graph by labelling sensitive vertices to depict high-level characteristics of Android apps. To determine which nodes are vulnerable, a malignant score is calculated. Then, to identify suspicious app patterns, a large number of sensitive subgraphs and their neighbour subgraphs are extracted. After removing the redundant graphs, the remaining graphs are embedded into a feature vector that represents each app. The model achieved a 97.04 malware detection rate as an F1 score.

Nguyen et al. [18] recommended using a technique known as PSI-Graph, which examines function-call graphs for each executable file to spot IoT botnets. When tested on 11,200 ELF files containing 7199 IoT botnet samples and 4001 benign samples, the method achieves 98.7% accuracy. Pektas et al. [19] utilized an API call graph to illustrate all possible malware execution paths. API call graphs are embedded as low-dimensional embeddings in deep networks. This research focuses on improving network performance by investigating various encoding algorithms and tuning network system parameters to achieve the best hyper-parameter combination and highest metric value. The suggested technique has an accuracy of 98.86%, an F-measure of 98.65%, a recall of 98.47%, and a precision of 98.84%. Kumar et al. [20] proposed a deep transfer learning-based method for malware image classification using ImageNet-trained CNN. Windows portable executable files (PEs) are transformed into grayscale images because related malware communities have similar visual attributes. After that, these visual features are fed into the customized deep CNN model. The method achieved 93.19% test accuracy for Microsoft datasets and 98.92% test accuracy for Malimg datasets. Vu et al. [7] suggested a malware classification method for encoding and organizing binary file bytes into images. The pixels in these evolved images are filled with space-filling curves and comprise statistical and syntactic features. The image-based features are then fed into the CNN model, which is used to classify malware. By incorporating entropy encoding and character class strategies, the proposed method achieved 93.01% accuracy on the Hilbert curve. A more powerful malware detection and classification system can be developed by combining the control flow and visual features.

## Proposed method

**Fig. 2:**
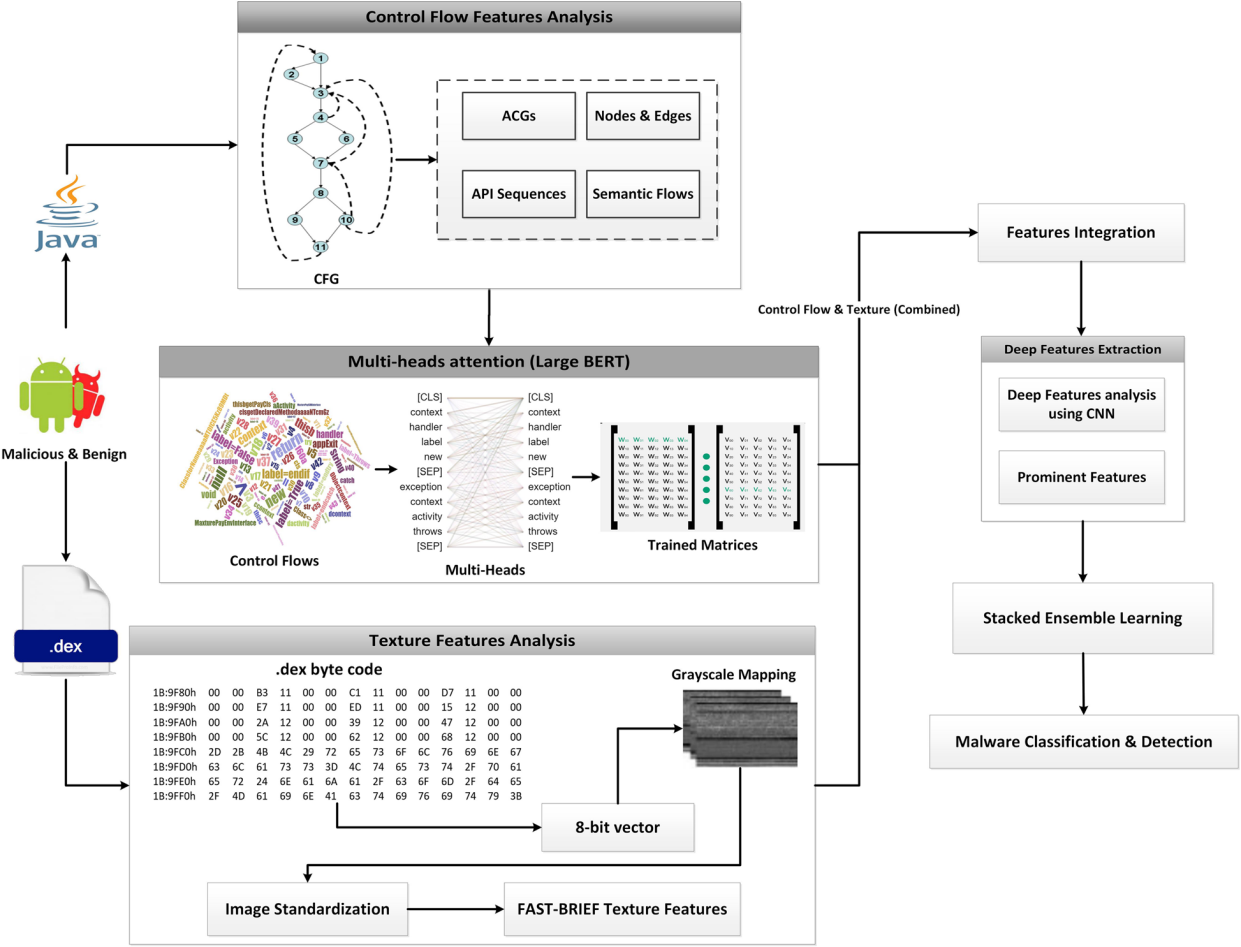
Malware detection system using ACGs-based multi-heads attention and image representation

Figure 2 depicts the proposed method for classifying Android malware, which combines ACGs with texture features. Reverse engineering tools are used to extract Java source codes and DEX files from Android APKs. To extract CFG features from Java code, the graph-based method is used. When dealing with malware detection, these are high-level features that should be traversed each time. As a result, instead of using complete CFG features, this method focused on ACGs features that can reduce execution load and extract more specific features. Following that, ACGs and texture features are extracted from Java source code and DEX files for effective malware detection and classification. The detailed steps are explained in the following sections.

### Reverse engineering of APKs

**Fig. 3:**
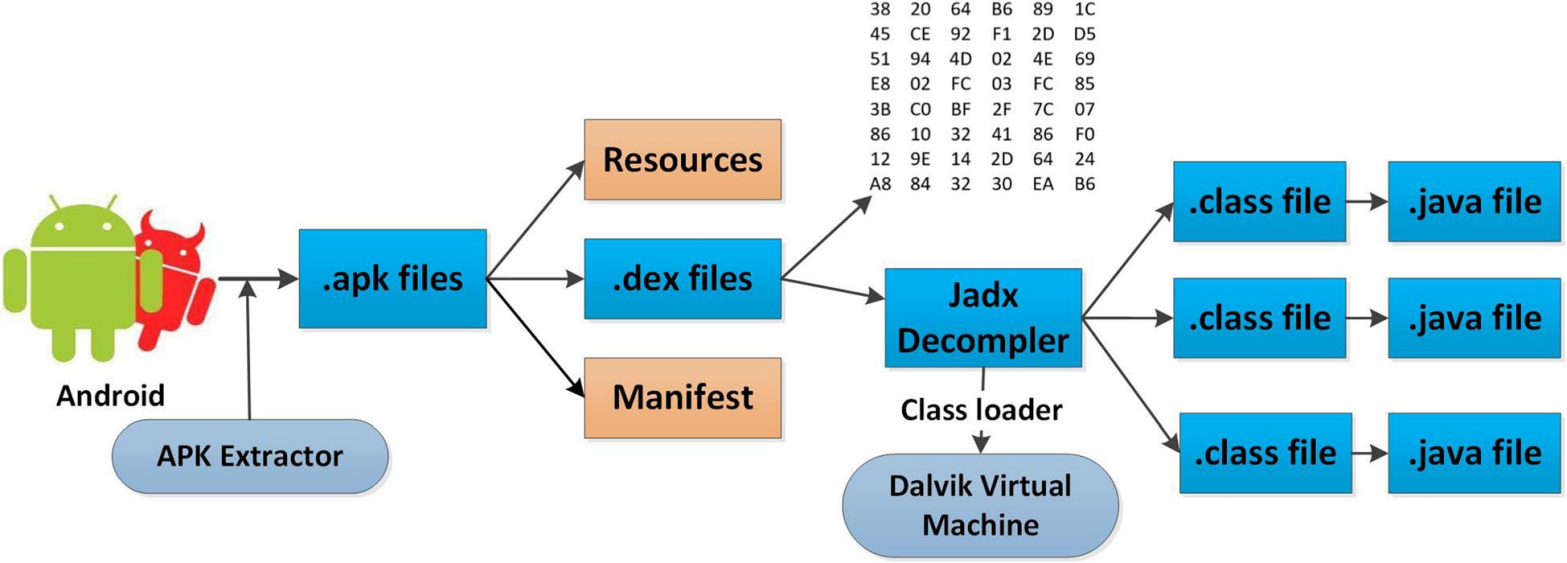
Reverse Engineering of Android APKs for hunting of Dex file and Java sources codes

Figure 3 depicts the reverse engineering procedure for retrieving Java codes and DEX files. To reverse-engineer the application, we would need its APK. The APK Extractor file explorer is used to open the extracted APKs folder in the Internal Storage directory. The chosen APKs are copied to system storage so they can be further processed. These APKs are then reversed to reveal the code. This can help us understand the structure of the code and identify the security measures they have implemented to avoid a reverse engineering attack. The [app].apk file is renamed to [app].zip and then unzip it up and retrieve it. The classes.dex file, which includes the app code, can be found within the retrieved repository. A Dalvik Executable, or DEX file, is an executable file that runs on the Android OS and contains the compiled script. The Jadx decompiler is then used to decompile the DEX file to extract the Java codes. In the proposed work, the java programming codes and DEX files are used together to extract features [21]. The reverse engineering process is shown in Algorithm 1.

**Algorithm 1:**
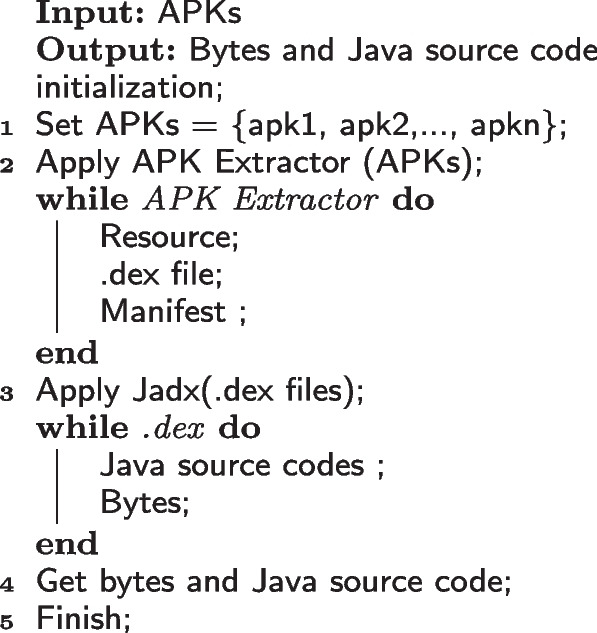
Reverse engineering of APKs

Further, we found URLs in Java codes that can be used to detect malicious activities as shown in Table 1. These URLs highlight interactions transmitted to the advertising network. To monitor the apps that are used and distribute related ads to the device, advertising companies gather this data to build a profile for the device. An individual phone can be tracked using the UDID, which is a special identification number.

**Table 1:** Malicious URLs found in Java source codes (adware APKs)

Source	URL
airpush	http://api.airpush.com/api.php
airpush	http://api.airpush.com/model/user/getappinfo.php?packageName=
airpush	http://api.airpush.com/redirect.php?market=
airpush	http://api.airpush.com/testicon.php
airpush	http://api.airpush.com/testmsg2.php
airpush	http://api.airpush.com/v2/api.php
airpush	http://api.airpush.com/v2/api.php?apikey=
adwhirl	http://cus.adwhirl.com/custom.php?appid=%s&nid=%s&uuid=%s&country_code=
adwhirl	http://met.adwhirl.com/exclick.php?appid=%s&nid=%s&type=%d&uuid=
adwhirl	http://met.adwhirl.com/exmet.php?appid=%s&nid=%s&type=%d&uuid=
adwhirl	http://cus.adwhirl.com/custom.php?appid=%s&nid=%s&uuid=

### Graph-based features analysis

**Fig. 4:**
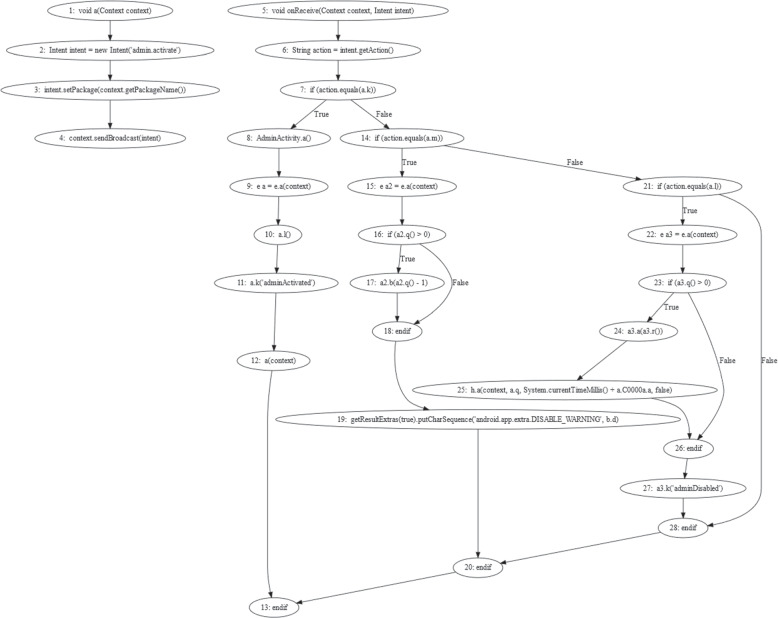
Control Flow Graph of ewind family of adware

The CFG includes graph-based features for converting Java code via logical paths. These paths are then used to represent the semantics of codes. It uses graph numerals to represent all paths that a code may take during implementation. Each node in the graph represents a basic block, such as a code statement that may or may not include a jump. The jump highlights the start and end of a block of statements. The directed edges represent the Java code execution order. As a result, the combined effect of nodes and edges represents the code layout. In a general sense, there are two blocks: the input block, which regulates the start of the flow, and the departure block, which leaves the control flow [4, 22, 23]. For a given Android app, this technique employs an ACG to interpret the global behaviour of the app via CFG. Considering that system APIs rather than developer-defined functions are always employed to invoke access to users’ data and system resource management. Malicious behaviour should always involve structured APIs. As a result, the ACG generation component aims to consciously tag specific APIs to describe malicious activities. Rather than employing a heuristic approach to examining key characteristics of malware functionality, we examined a large volume of benign and malware apps and converted each app into the appropriate ACG. A CFG is a directed graph $G = (N,\ E,\ entry)$ as shown in Fig. 4, where N is the node set and each API in the code (such as system API and user-defined API) denotes one node; $E = \{ n1 > n2\ |n1,\ n2 \in N$ and *n*2 may be immediately executed after $n1\}$; and entry is the entry point as a node. Because every App has an entry function called *main*(), we can consider *main*() to be the entry point. The Java code is examined further to identify malicious APIs. Malicious apps use threatening APIs to achieve their objectives. For instance, obtaining personal information using SMS APIs and sending it through a subnet with network APIs can cause confidentiality leaks. Examining API usage can help identify this type of malware. Furthermore, some suspicious behaviour would make extensive use of standard APIs. For instance, if network APIs are frequently used, a DOS attack may happen. The frequency of APIs can thus be used to identify such types of malware. Although this type of evidence helps with malware detection, it is insufficiently precise. A benign application will use the same APIs infrequently throughout its lifetime, making it difficult to detect malware by evaluating recurrence. However, we can easily solve this problem by examining the API sequence. The logical execution paths of CFGs can be used to determine the order of APIs. Thus, by exploring the chronological API trend, we can learn about its behaviour. Table 2 shows some APIs found in the codes. These APIs can be used for malicious activities to control the functionalities of Android apps. For instance, “*android.permission.USE_CREDENTIALS*” API is used to steal the user credentials to get access to the genuine account over the Android app. Similarly, the “*android.permission.ACCESS_COARSE_LOCATION*” API can be used the access the location of the genuine user to further perform malicious activities.

**Table 2:** Malicious activities using different permission types in Java source codes (adware APKs)

Activity	API for different types of permission
Synchronization	android.permission.READ_SYNC_SETTINGS
uninstall shortcuts	com.android.launcher.permission.UNINSTALL_SHORTCUT
user credentials	android.permission.USE_CREDENTIALS
read settings	com.motorola.dlauncher.permission.READ_SETTINGS
access location	android.permission.ACCESS_COARSE_LOCATION
install shortcut	com.motorola.dlauncher.permission.INSTALL_SHORTCUT
Synchronization	android.permission.READ_SYNC_STATS
Synchronization	android.permission.WRITE_SYNC_SETTINGS
internet access	android.permission.INTERNET
vendor billing	com.android.vending.BILLING
install shortcut	com.lge.launcher.permission.INSTALL_SHORTCUT
send SMS	android.permission.SEND_SMS

### Transfer learning with multi-heads attention

We used the multi-head attention concept using the BERT-large model to extract train features from ACGs. A contextual model called BERT can produce multiple definitions for words in a sentence based on the relationships between those words [24, 25]. It is also recognized as a bidirectional model due to its ability to evaluate both the right and left contexts of words. Regardless of how the term appears in the document, non-contextual models produce only a word description. For instance, the phrase “match the words” and “light the match” can be viewed similarly to the word “match”. The ACGs features are organized into chronological patterns of APIs. The tokenization method is used to divide the ACGs into small features while maintaining order. We used the multi-head attention concept with the BERT-large model to obtain train features from ACG features.

**Fig. 5:**
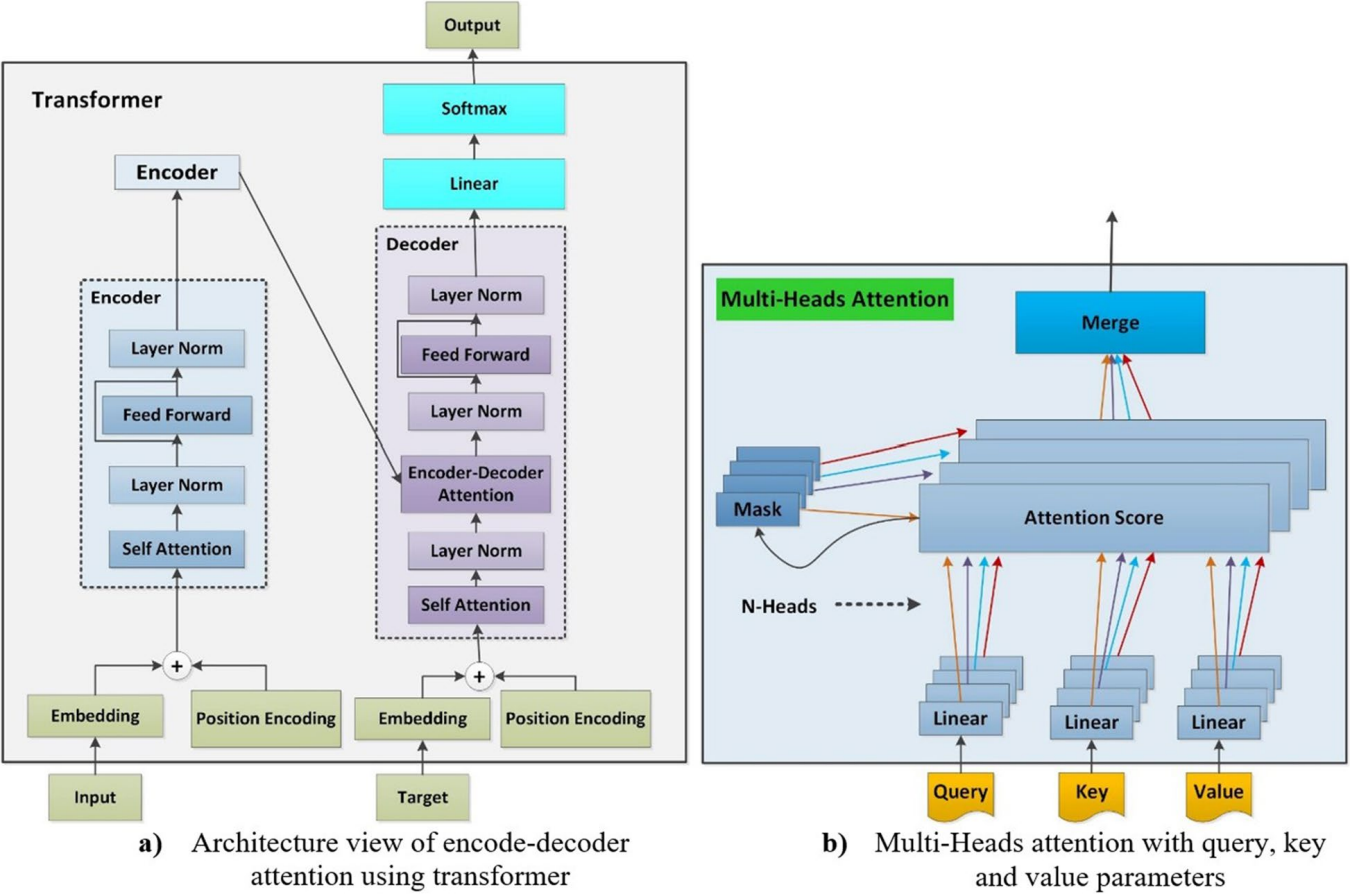
Deep view of multi-heads attentions using transformers

BERT-large has 24 encoded layers, 1024 hidden sizes, 16 self-attention heads, and 340M parameters. Figure 5(a) shows encoder and decoder transformer attention architecture. Embedding and positional encoding layers are used to encode and decode ACG features. The embedding layer stores API meaning. There are two embedding layers in the transformer. Input embedding receives the API sequence. After moving the target one stance to the right and putting a start token at the first location, the target sequence is fed to the second embedding layer. The embedding layer maps each API feature into an embedding vector, which is a richer depiction of ACGs. The position encoding is calculated separately from the API sequence. The embedding and position encoding layers work on API sequence matrices. The embedding uses an API-ID matrix (APIs, API sequence length). Each API-ID is encoded into an APIs vector whose length is the embedding size, resulting in an (APIs, API length, embedding size) output matrix. The position encoding employs the same encoding size as the embedding size. Therefore, it generates a matrix with a similar shape that can be incorporated into the embedding matrix. Each encoder in the encoder stack has a multi-head attention layer and feed-forward layer. Each decoder has two multi-head attention layers and a feed-forward layer. Linear and softmax layers are output.

Figure 5(b) depicts the iterative and simultaneous computations performed by the attention module of the transformer. Each of these is known as an attention head. The N-way split of the query, key, and value parameters is handled by a separate head thanks to the attention module. The sum of all of these related attention computations results in the final attention score. This is known as “multi-head attention”, and it improves the transformer’s capacity to encode relationship dynamics and refinement for each ACG feature. The procedure for extracting train textual features from ACGs is shown in Algorithm 2.

**Algorithm 2:**
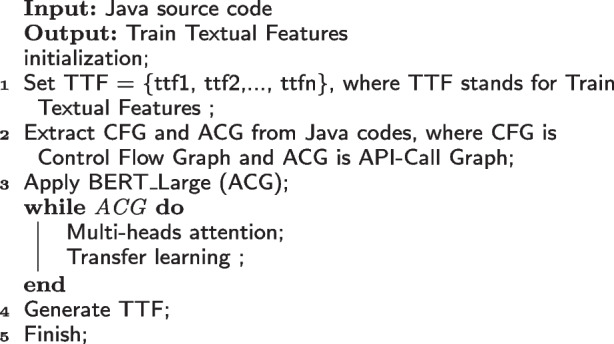
Generating TTF from ACGs

### Malware visualization and texture feature extraction

**Fig. 6:**
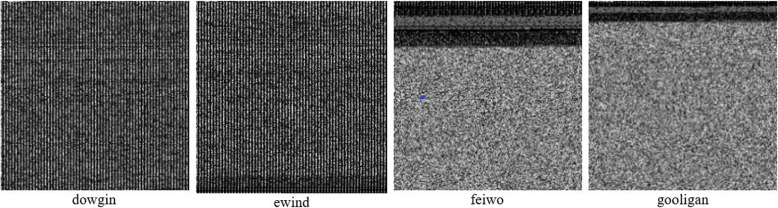
Malware images extracted from adware families with size 256x256

We investigated a malware detection approach based on texture features because it is frequently modified to avoid static and dynamic analysis. This approach can successfully counter anti-detection techniques like dynamic feature extraction obfuscation and fingerprint exploitation. The DEX file contains the byte streams that demonstrate the correct APK structure. We developed a method that converts the byte streams to grayscale images using an 8-bit vector. Following this, all image dimensions are set to 256x256 pixels. Figure 6 depicts images with a resolution of 256x256 extracted from the DEX files of adware families such as dowgin, ewind, feiwo, and gooligan. It is found that a large DEX size is shrunk down to a more manageable one. For instance, the DEX in the image is reduced from megabytes to kilobytes. Consequently, it may be feasible to decrease computational resources.

By combining FAST, and BRIEF, texture features are then retrieved from DEX images [26]. The FAST extractor can perform calculations quickly and accurately. First, it detects edges by circling a pixel (p) with 1 to 16 pixels known as the Bresenham circle. Pixels from 1 to 16 are now identified. Examine a random sample of N labels inside the circle to see if any of them correspond to pixels that are brighter than the 16 chosen pixels. Because BRIEF is only a feature descriptor, features are extracted and described using the FAST corner extractor. For ease of use, the implementation procedure is divided into three phases.

- The image is first loaded into memory.
- A copy of the image is generated that is identical in terms of scaling and rotation.
- The combination of the BRIEF descriptor and FAST extractor is used to highlight features.

Algorithm 3 depicts the extraction of texture features.

**Algorithm 3:**
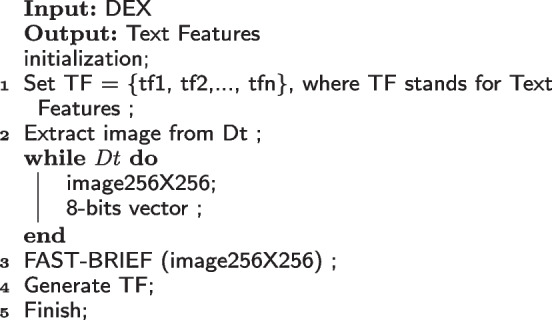
Generating texture features

### Deep features selection

The ACGs and texture features are combined for effective malware detection and classification. The CNN network is made to mine a wide range of attributes and extract significant and in-depth traits. This can lighten the burden and processing capability required by the classification model. To achieve this, the merged features (ACGs, texture) are fed into CNN. CNN model performs better with diverse data types, including textual, texture, and media features [27, 28]. For this purpose, we employ 1-D convolutional layers, pooling layers, dropout layers, and a fully connected layer. Convolution functions as a filter, commuting via the selected features continually to select the most suitable embeddings. A feature map, which is created by each filter, contains a clean set of features. The hyper-parameters tunning is used to find the best number of filters. Four convolution layers are being used, each with 64, 128, 256, and 512 filters. Max-pooling lessens the feature space size, feature variety, and model complexity. The most significant features from the previous set are also generated as a feature map by this layer. Additionally, we add a Keras batch normalization layer to the CNN network for improved performance. Batch normalization ensures that the resultant mean remains close to zero while maintaining a standard deviation that is close to one. Particularly, its behaviour changes between training and testing. This helps keep the learning process steady and cuts down on the time required to train the model. Overfitting is addressed by the dropout and softmax layers in the proposed CNN network. The yield of the CNN model is represented by Eq. 1.

1 $$\begin{aligned} o^{1}_{k}=f\left(c^{1}_{k} \sum \limits ^{N_{l-1}}_{i=1} Con1D\left(X^{l-1}_{ik},t^{l-1}_{i}\right) \right) \end{aligned}$$

where $c^{1}_{k}$ is the parameter bias of the kth neuron in the first layer, $t^{l-1}_{i}$ is the outcome of the $i^{th}$ neuron in layer $l-1$, $X^{l-1}_{ik}$ is the kernel strength from the $i^{th}$ neuron in layer $l-1$ to the $k^{th}$ neurons in layer l, and *f*() is the activation function. After analyzing the deep features, we chose the top 250 prominent features for accurate malware classification.

### Stacked generalization ensemble learning

**Fig. 7:**
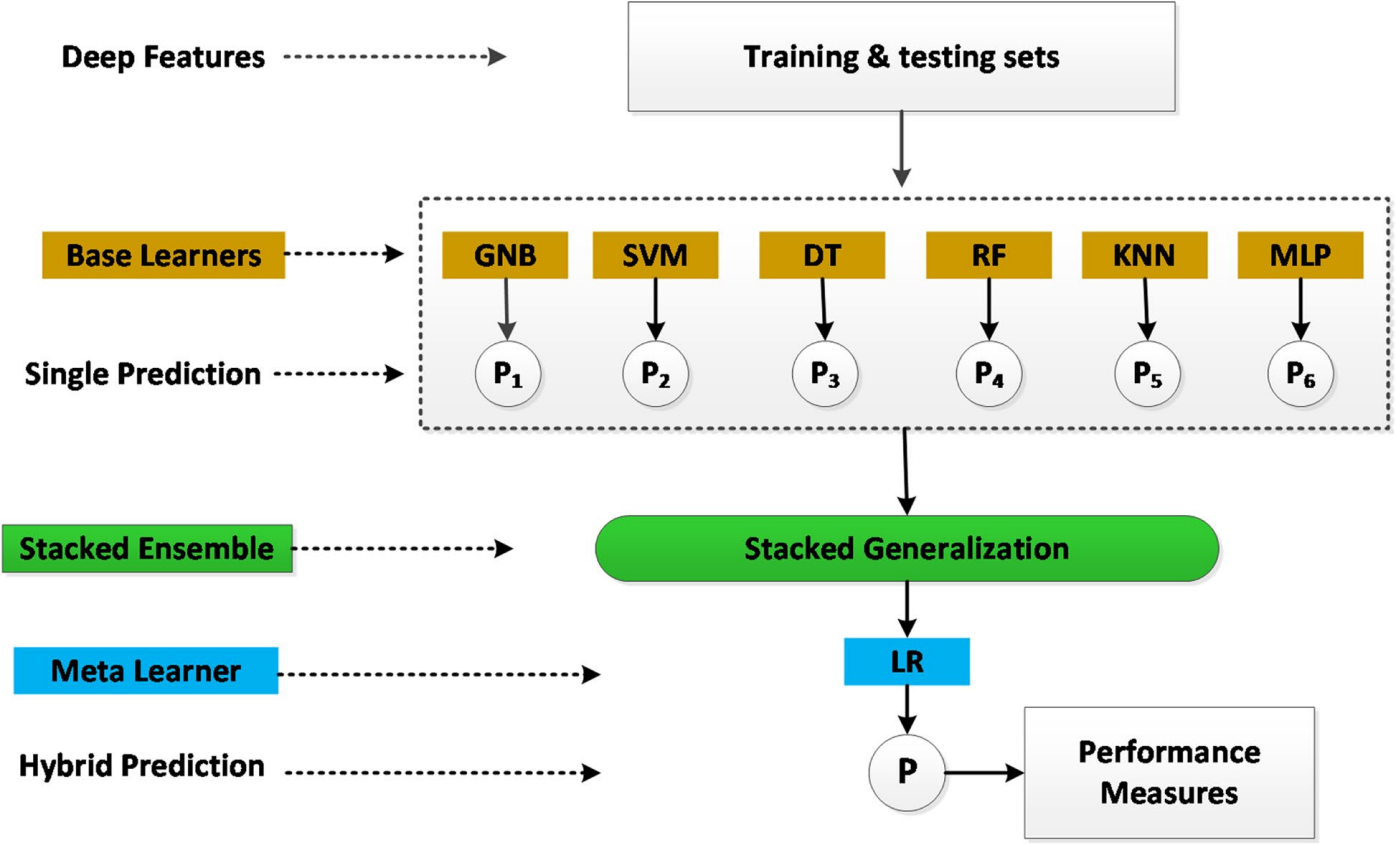
Stacked ensemble learning using generalization concept for malware classification

Stacked Generalization has a multilevel framework that has been thoroughly examined and implemented for several machine learning problems [29, 30]. The contributions of each sub-model to the combined prediction can be weighted, which can improve model averaging for malware detection. This can be extended by learning a completely new model to determine the best way to combine the contributions of several sub-models. This procedure is known as a stacked generalization ensemble, and it can outperform the predictive abilities of any single model. We develop a stacked generalized ensemble model to weigh the contributions of each sub-model to the joint prediction according to the expected output of the sub-models as shown in Fig. 7. Individual learners are the level-0 learners, and the combiner is the level-1 learner. Following is specific information regarding the stacked generalization.

1. Level-0: This is also known as base-learner. The deep features are divided into training and testing sets, and the training set is then used to generate base learners via base learning models. We combine several models to work as a base-learner, including Gaussian Naïve Bayes (GNB), Support Vector Machine (SVM) with Radial Basis Function (RBF), Decision Tree (DT), Random Forest (RF), K-Nearest Neighbor (KNN), and Multi-Layer Perceptron (MLP). Using out-of-sample data, the prediction is made for each base learner.
2. Level-1: This is also known as meta-learner. The outcome of the base learners is fed into the meta learner’s data, and a single meta-learner learns to make accurate malware detection from this data. We used Logistic Regression (LR) as a metal learner. To prevent overfitting, the meta-learner is trained on a different dataset than the instances used to train the base learners. The testing part of the deep features is used to train the metal learner.

When compared to individual models, we achieve better malware detection and classification results. It is capable of optimizing the best linear combinations of models. This enables us to obtain the optimal blend of diversity from each model and achieve the highest level of detection accuracy. However, the computation time for a stacked ensemble is longer than for any single model. Algorithm 4 depicts the process of detecting malware using hybrid features.

**Algorithm 4:**
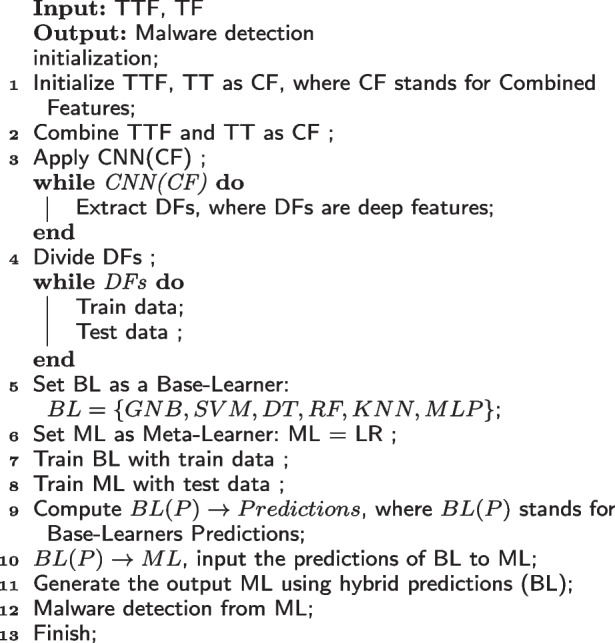
Malware detection

## Results and discussions

### Dataset preparation

We prepared a customized dataset from CIC-InvesAnd-Mal2019 [31] by using reverse engineering and data mining tools. Originally, the dataset is available in the form of APKs. It includes four types of malware such as adware, ransomware, scareware, and SMS. Each malware type is further subdivided into 10 to 11 families. This dataset is been compiled to install 5, 000 samples on real Android devices. These samples originated from 42 distinct families of 342 malicious Android apps as shown in Table 3. These APKs are thoroughly analyzed to unbox and prepare our customized dataset for effective malware detection, as shown in Table 4. The Java programming codes and DEX files are obtained by reverse engineering the Android APKs. There are approximately 3.2K ACGs collected from adware and ransomware, and 3.4K ACGs collected from scareware and SMS, respectively. Similarly, the proposed method crawls the train and texture features with 8.4K for both adware and ransomware and 8.6K for scareware and SMS. These features are combined further to extract deep features for improved malware classification results.

**Table 3:** CIC-InvesAndMal2019 dataset

APK	Type of families	Description
Adware	Dowgin, Ewind, Feiwo, Gooligan, Kemoge, koodous, Mobidash, Selfmite, Shuanet, Youmi	Adware is an unwanted app that displays ads in your browser.
Ransomware	Charger, Jisut, Koler, LockerPin, Simplocker, Pletor, PornDroid, RansomBO, Svpeng, WannaLocker	A malicious app that threatens to block data or a device until the suspect pays a ransom.
Scareware	AndroidDefender, AndroidSpy, AV, AVpass, FakeApp, FakeApp.AL, FakeAV, FakeJobOffer, FakeTaoBao, Penetho, and VirusShield	Scares people into visiting fake or infected websites or downloading malicious files.
SMS	BeanBot, Biige, FakeInst, FakeMart, FakeNotify, Jifake, Mazarbot, Nandrobox, Plankton, SMSsniffer, Zsone	It is a mobile text messaging-based phishing cybersecurity attack.
Benign	—	Clean apps (Not malicious)

**Table 4:** Our customized dataset prepared from CIC-InvesAndMal2019

APK	No. of ACGs	Train Features (APIs)	Texture Features	Combined
Adware	$\sim 3.2K$	$\sim 8.4K$	$\sim 27K$	$\sim 35.4K$
Ransomware	$\sim 3.2K$	$\sim 8.4K$	$\sim 27K$	$\sim 35.4K$
Scareware	$\sim 3.4K$	$\sim 8.6K$	$\sim 28K$	$\sim 36.6K$
SMS	$\sim 3.4K$	$\sim 8.6K$	$\sim 28K$	$\sim 36.6K$
Benign	$\sim 4.8K$	$\sim 12.6K$	$\sim 40.4K$	$\sim 53K$

### Performance indicators

We chose an 80% to 20% training to testing ratio, which is a widely used benchmark. We employed six different types of evaluation metrics: precision, recall, F1-score, Matthews Correlation Coefficient (MCC), accuracy, and confusion matrix. The proportion of malware and benign apps are correctly classified as True Positives (TPs) and True Negatives (TNs). Likewise, the number of malware and benign apps are incorrectly classified as False Positives (FPs) and False Negatives (FNs). An accuracy matrix is used to evaluate general classification performance. This equals the sum of instances that have been correctly classified divided by the total number of instances. The MCC measures the degree of correlation between expected and actual values. It can produce a value ranging from $-1$ to $+1$. The MCC can be $+1$ when the predictions are correct, and 0 when it performs no better than a random prediction. Furthermore, the MCC can be $-1$ when predictions and observations disagree. Equations 2 to 6 show the evaluation matrices.

2 $$\begin{aligned} TPR=\frac{TP}{TP+FN};\ FPR=Recall=\frac{FP}{FP+TN} \end{aligned}$$

3 $$\begin{aligned} Precision= \frac{TP}{TP+FP} \end{aligned}$$

4 $$\begin{aligned} F_{1}-Score= \frac{2*TP}{2TP+FP+FN} \end{aligned}$$

5 $$\begin{aligned} Accuracy= \frac{TP+TN}{TP+TN+FP+FN} \end{aligned}$$

6 $$\begin{aligned} MCC= \frac{TPXTN-FPXFN}{\sqrt{(\tau _{1})(\tau _{2})(\tau _{3})(\tau _{4})}} \end{aligned}$$

Where $$\tau _{1}= (TP+FP),\ \tau _{2}=\ (TP+FN),\ \tau _{3}=(TN+FP),\ \tau _{4}= (TN+FN)$$.

### Results analysis

**Fig. 8:**
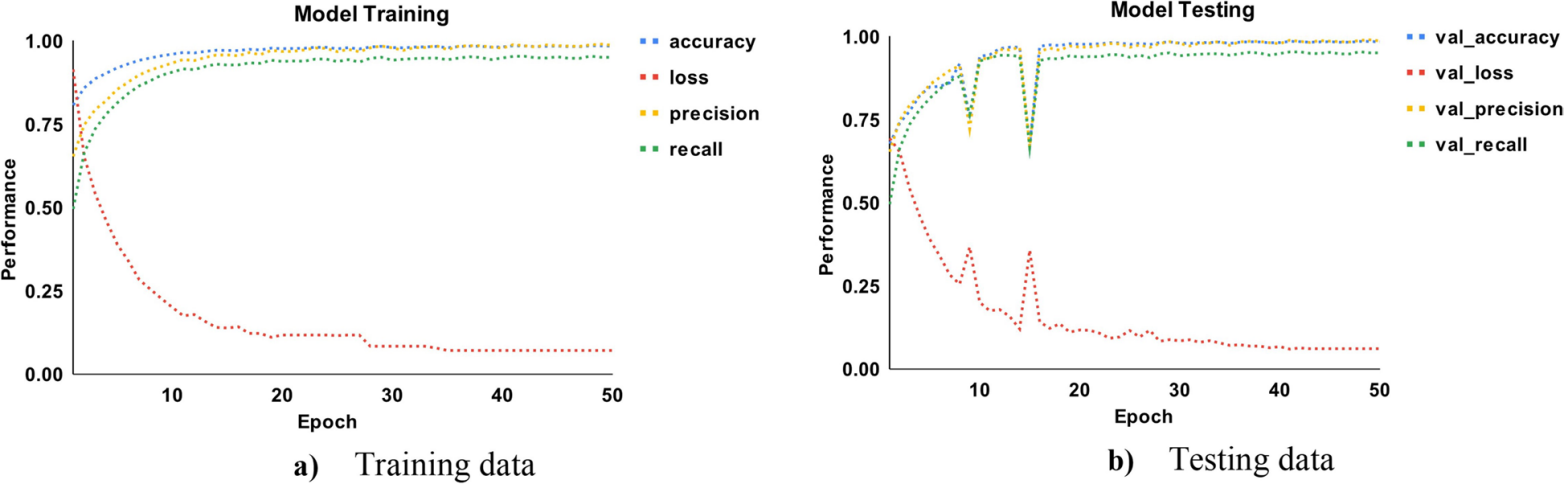
Epoch curves for training and testing data points for malware detection (accuracy, loss, precision, recall)

These epoch curves can be used to demonstrate the dynamic behaviour of the model during training on each epoch for malware detection and classification. Figure 8 shows the training and testing epoch curves for malware detection using accuracy, loss, precision, and recall. The colours blue, red, orange, and green represent the accuracy, loss, precision, and recall, respectively. Using the training data in part a, the accuracy begins at 80% and increases to 99% by the 20th epoch. The loss begins at 97% and gradually decreases with each epoch. The loss is approximately 5% on the 28th epoch and then becomes more or less constant. Similarly, precision and recall begin at 70% and 50%, respectively, and gradually increase to 98% in the 20th epoch. The inverse relationship between accuracy and loss indicates that the proposed model performs better on training data. Part b also shows that the accuracy and loss are inversely proportional, indicating that the model performs better on test data. In the 15th epoch, there is a drop of up to 70% in accuracy, precision, and recall, with the loss increasing to 29%. Overall, the three performance measures provide 99% performance on the 23rd epoch and are more or less constant after that. In addition, the normal behaviour of these dynamic curves indicates that there is a reduced likelihood of overfitting.

The comparison of the five malware detection performance measures is shown in Table 5. The KNN model has the lowest performance with (precision, recall, F1-score, MCC, and accuracy), (96%, 98%, 97%, 97.42%, and 97.12%), respectively. However, the proposed ensemble model performs best in terms of (precision, recall, F1-score, MCC, and accuracy), with (99%, 99%, 99%, 99.14%, and 99.27%). While the MLP comes in second place after the ensemble. When compared to the base learners, the stacked ensemble as meta learner performs the best. Table 6 shows the performance compassion for malware detection for both malware and benign class. The precision, recall, and F1-score for each class are presented using the base learner and meta learner. The stacked ensemble performed the best, with (100%, 98%, and 98%) for malware and (97%, 99%, and 99%), respectively. While the KNN performs the worst for malware and benign.

**Table 5:** Comparison of performance measures for malware detection

Model	Precision (%)	Recall (%)	F1-score (%)	MCC (%)	Accuracy (%)
GNB	98	98	97	97.84	98.13
SVM-rbf	97	98	98	97.62	98.06
DT	97	98	98	97.77	98.15
LR	98	98	98	97.82	98.18
RF	97	98	97	96.74	97.82
KNN	96	98	97	97.42	97.12
MLP	97	98	98	98.08	98.22
Ensemble	99	99	99	99.14	99.27

**Table 6:** Per-class comparison of performance measures for malware detection

Model	App	Precision (%)	Recall (%)	F1-score (%)
GNB	Malware	100	97	98
	Benign	100	96	100
SVM-rbf	Malware	100	97	98
	Benign	96	100	98
DT	Malware	98	99	98
	Benign	99	97	98
LR	Malware	100	97	98
	Benign	96	100	98
RF	Malware	97	98	97
	Benign	98	96	97
KNN	Malware	97	98	98
	Benign	98	96	97
MLP	Malware	97	100	98
	Benign	100	96	97
Ensemble	Malware	100	98	98
	Benign	97	99	99

**Fig. 9:**
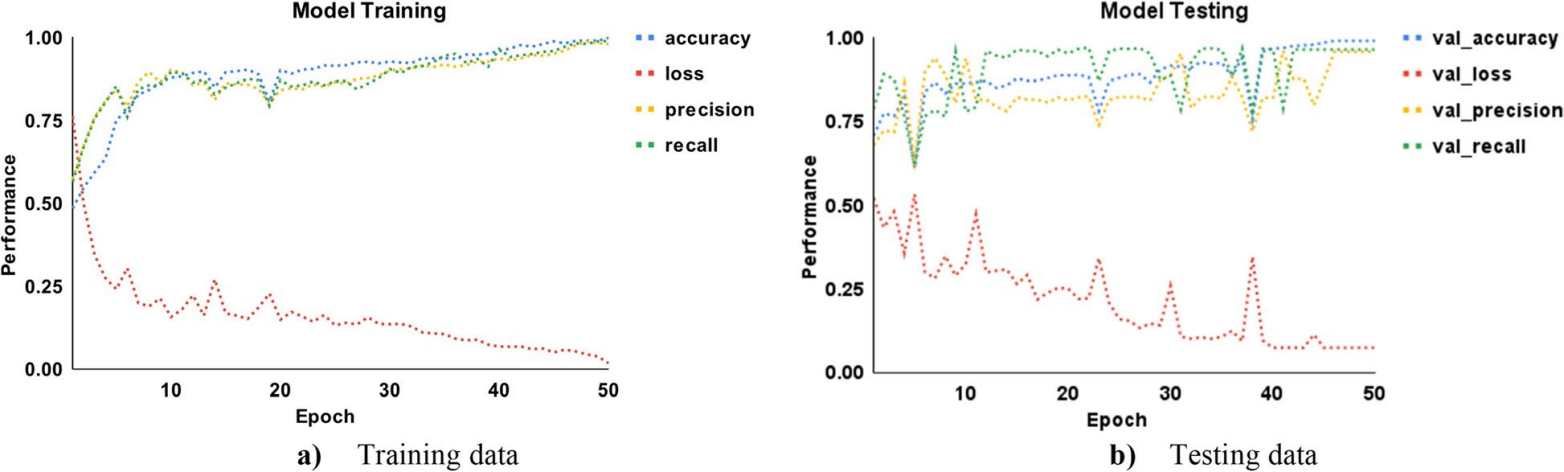
Epoch curves for training and testing data points for malware classification (accuracy, loss, precision, recall)

Figure 9 shows the training and testing epoch curves for malware classification using accuracy, loss, precision, and recall. In part a using training data, the accuracy curve starts from 50% and gradually increases to reach 83% on the 20th epoch. Further, it moves up and reaches 98% in the 40th epoch. After that, it is more or less constant. Conversely, the loss starts from 75% and then drops gradually up to 20% in the 22nd epoch. Further, it is more or less constant after the 40th epoch and drops up to 4%. The precision and recall behave close to accuracy which indicates that the proposed approach performs better for training data. In part b, the same performance measures are shown for testing data. The accuracy, precision, and recall behave abruptly sometimes but provide the best performance. There is a slight drop up to 75% and an increase in loss up to 32%, but after that, they behave normally. The performance comparison for malware classification is shown in Table 7. The ensemble provides the best classification results, with precision, recall, F1-score, MCC, and accuracy of 100%, 98%, 98%, 98.52%, and 99.17%, respectively. While the SVM-rbf achieves the lowest classification performance.

**Table 7:** Comparison of performance measures for malware classification

Model	Precision (%)	Recall (%)	F1-score (%)	MCC (%)	Accuracy (%)
GNB	98	97	97	97.16	97.41
SVM-rbf	94	94	94	91.40	93.56
DT	95	95	95	93.91	94.71
LR	98	97	97	96.59	97.42
RF	97	97	97	96.21	97.16
KNN	97	97	97	96.86	97.02
MLP	98	97	98	97.81	98.37
Ensemble	100	98	98	98.52	99.17

**Fig. 10:**
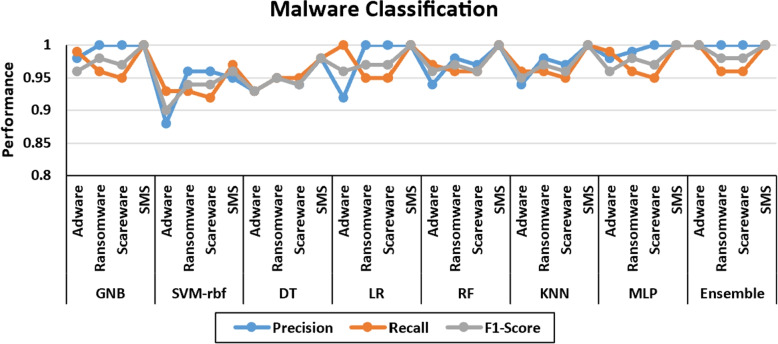
Per-class comparison of performance measures for malware classification

**Fig. 11:**
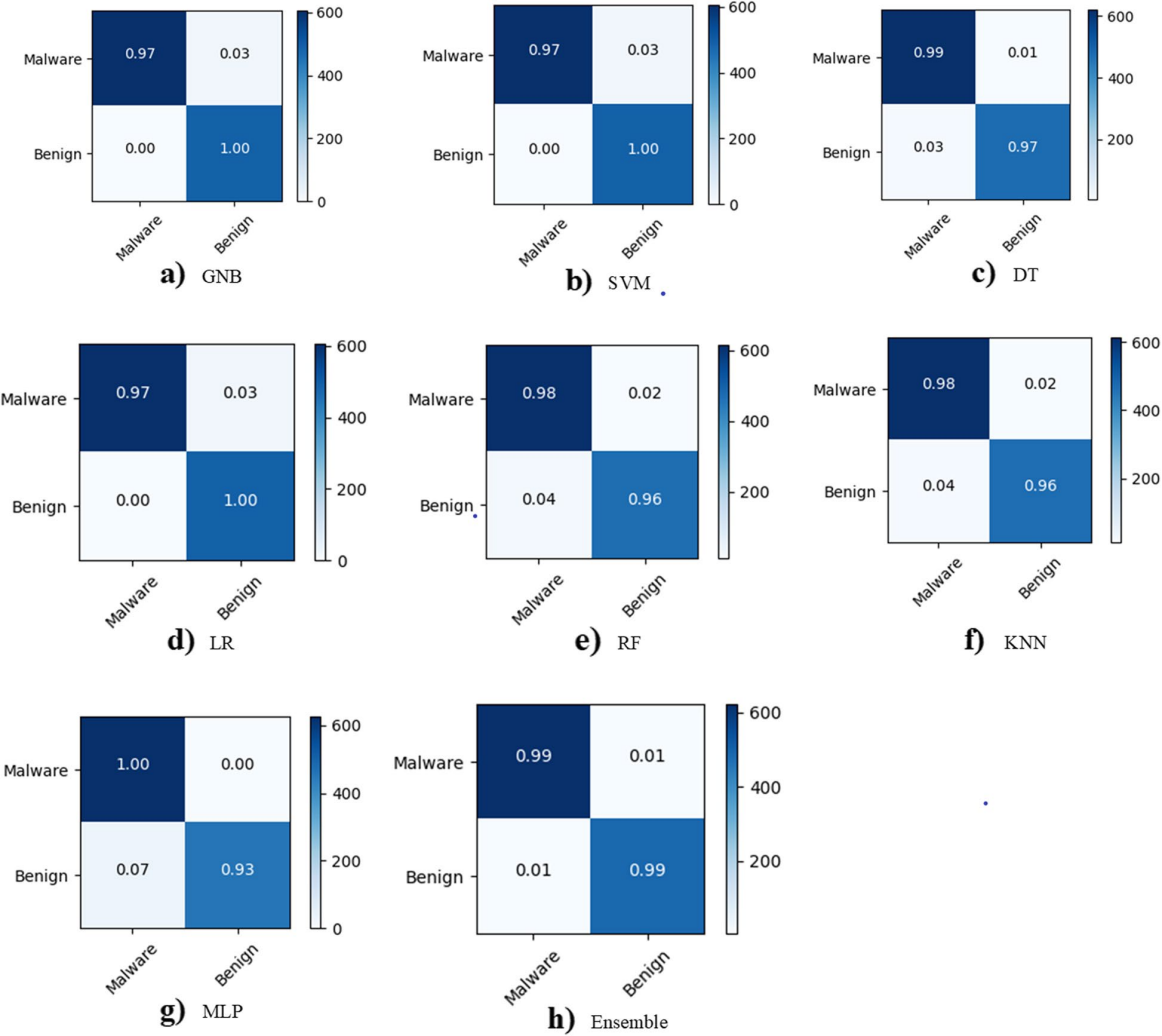
Comparison of classification/misclassification using confusion matrices for malware detection

**Fig. 12:**
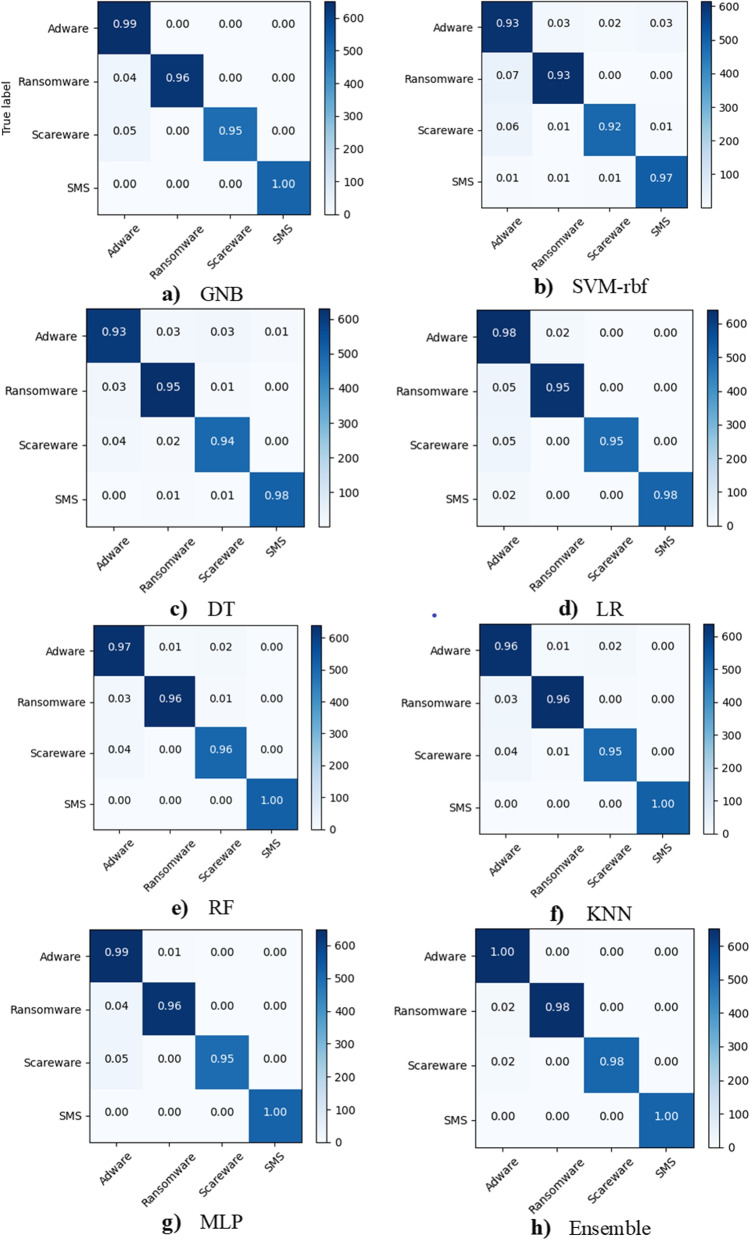
Comparison of classification/misclassification using Confusion matrices for malware classification

Figure 10 depicts the malware classification for each type of malware, namely adware, ransomware, scareware, and SMS. The precision, recall, and F1-score are indicated by the blue, orange, and gray colours. The recall is lowest when using base and meta learners, while the F1-score is the best. However, accuracy yields the best results for ransomware and scareware when using ensemble, while it yields the worst results for adware when using LR and SVM-rbf. There is a drop in accuracy and F1-score of up to 84% when using SVM-rbf for adware, indicating that this base learner provides the worst classification results. The ensemble produces the best results overall. Figure 11 depicts the confusion matrices, which can be used to investigate classification and misclassification for malware detection. The confusion matrix is provided for each base learner and ensemble. The blue diagonal values represent classification values, while the off-diagonal values represent misclassification. The ensemble model produces the best classification results of 99%, 99%, and 1%, 1% for malware and benign, respectively. Figure 12 depicts the confusion matrices for malware classification. It is once again demonstrated that SVM-rbf has the lowest performance while the ensemble has the highest. For instance, the classification results for adware, ransomware, scareware, and SMS are 93%, 93%, 92%, and 97%, respectively, whereas the ensemble has 100%, 98%, 98%, and 100% for the same classes. It is shown that the proposed hybrid results using the ensemble model outperform the base learners for each malware variant.

**Fig. 13:**
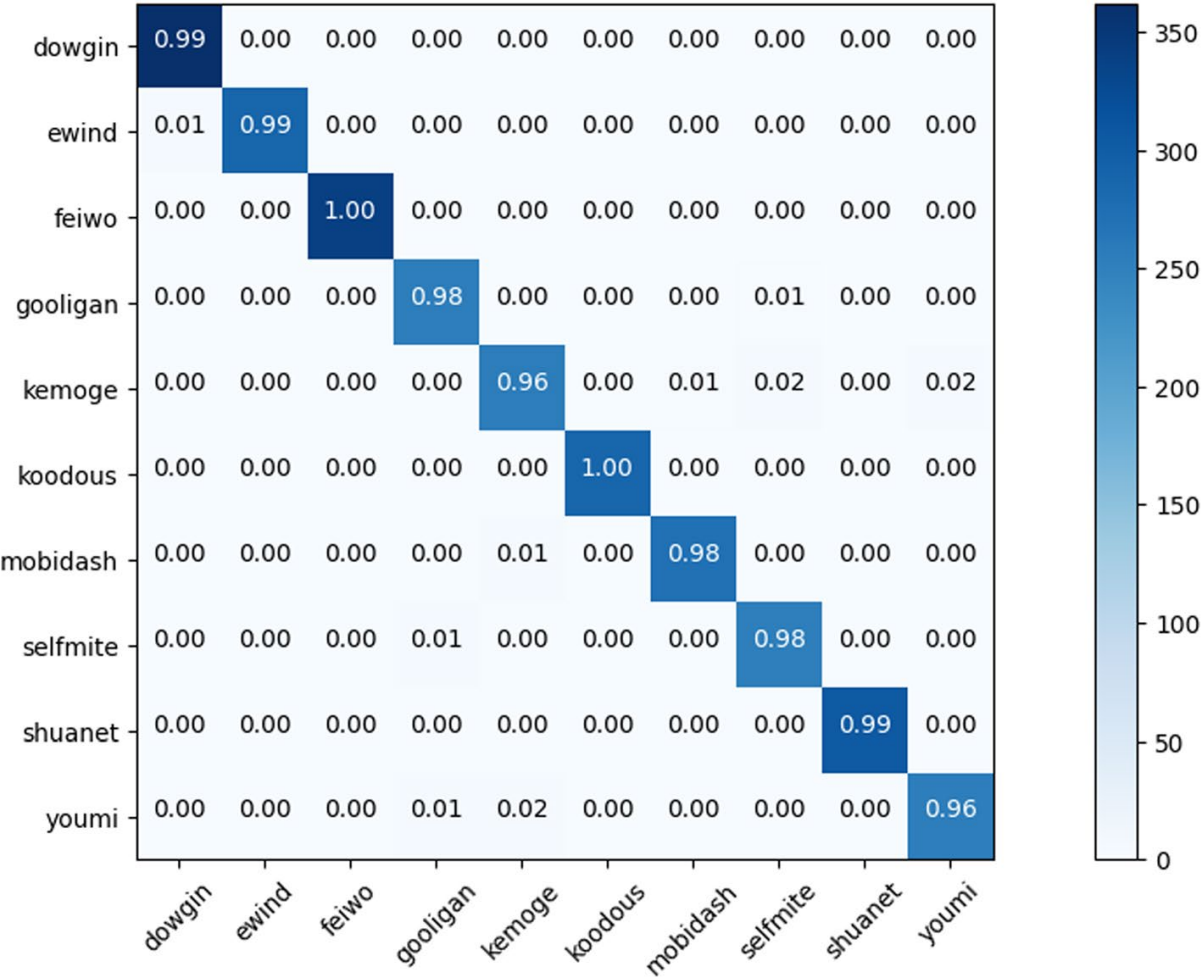
Confusion matrix for adware families using ensemble

To dig deeper, we examined the classification results for each adware family. Table 8 summarizes the performance of the proposed approach for adware families, which include dowgin, ewind, feiwo, gooligan, kemoge, koodous, mobidash, selfmite, shuanet, and youmi. When compared to others, the feiwo, kudous, and shuanet have the best classification results. For feiwo, kudous, and shuanet, the precision, recall, and f-score are (99%, 100%, 100%), (100%, 100%, 100%), and (100%, 99%, 100%), respectively. However, kemoge and youmi produce the fewest results. For instance, the precision, recall, and F1-score for kemoge and youmi are (97%, 96%, 96%), (98%, 96%, 97%), and (98%, 96%, 97%), respectively. Figure 13 depicts the confusion matrix using the ensemble model, which shows the classification and misclassification values for each adware family. The results for adware are then generated by averaging the classification results from each family of adware. As a result, each family successfully fulfills its function for the parent type of malware.

**Table 8:** Comparison of performance measures for malware family classification

Family	Precision (%)	Recall (%)	F1-score (%)
dowgin	98	99	99
ewind	99	99	99
feiwo	99	100	100
gooligan	97	98	98
kemoge	97	96	96
koodous	100	100	100
mobidash	99	98	98
selfmite	97	98	98
shuanet	100	99	100
youmi	98	96	97

### Comparison with other methods

**Fig. 14:**
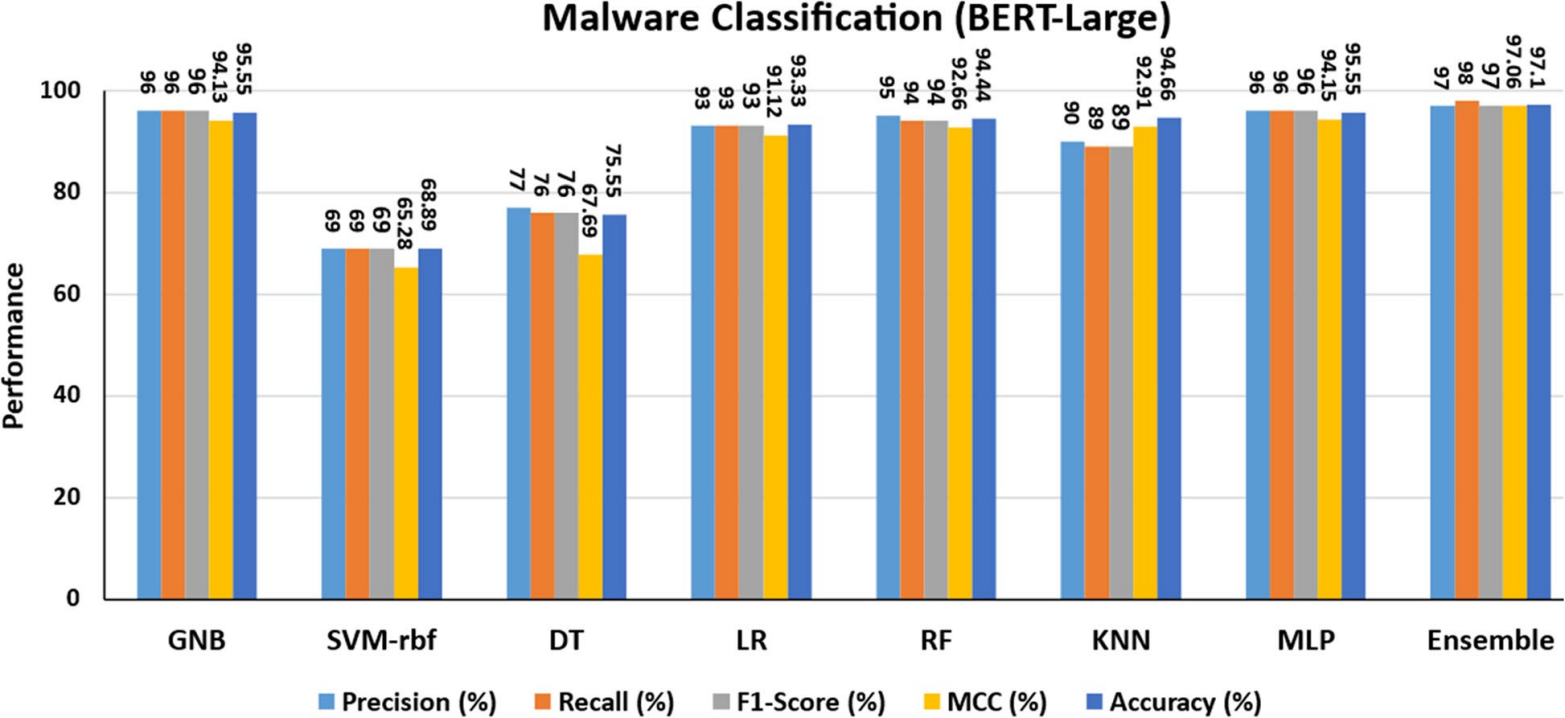
Malware classification using BERT-large (without texture features)

To demonstrate the effectiveness comprehensively, the proposed method is compared to other methods. The proposed method used a hybrid approach that combined ACGs and texture features. To classify malware, we only used ACG features and the BERT large model as shown in Fig. 14. Light blue, orange, grey, yellow, and dark blue represent precision, recall, F1-score, MCC, and accuracy, respectively. The SVM-rbf produces lower classification results for precision, recall, F1-score, MCC, and accuracy, which are 69%, 69%, 69%, 65.28%, and 68.89%, respectively. While the ensemble model provides good classification results (97%, 98%, 97%, 97.06%, and 97.1%), Because we are only using ACGs features, the results are lower than the hybrid features. As a result, it is demonstrated that hybrid features produce the best classification results. Furthermore, the proposed approach is compared to cutting-edge transfer learning approaches. Table 9 exhibits the performance of various trained models, namely word2vevc, BERT-base, and BERT-large. First, these trained models are used for malware classification without the texture feature. The same models are then combined with texture features to classify malware to demonstrate the effectiveness of the combined approach. Using textual features with the word2vec model yields the poorest results. BERT outperforms models such as word2vec. According to word2vec, every word has the same representation, even though the context in which a word appears can completely change its meaning. BERT generates dynamically influenced word representations based on neighbouring words. For instance, using word2vec, the precision, recall, F1-score, MCC, and accuracy are 95%, 95%, 96%, 95.12%, and 95.61%, respectively. The BERT-large performs better than the BERT-base when only textual features are used. Overall, the hybrid approach using BERT-large and texture features produces the best classification results when compared to other transfer learning approaches.

**Table 9:** Performance comparison of transfer learning methods with the proposed approach

Method	Precision (%)	Recall (%)	F1-score (%)	MCC (%)	Accuracy (%)
word2vec	95	95	96	95.12	95.61
BERT-base	96	97	96	96.21	96.34
BERT-large	97	98	97	97.06	97.12
Texture with word2vec	98	98	99	98.13	98.38
Texture with BERT-base	98	99	98	98.21	98.52
Texture with BERT-large	99	99	99	99.14	99.27

Table 10 shows the performance comparisons with related and recently published research. Arslan et al. [15] proposed to create a graphical Android malware detection tool. The features of Androidmanifest.xml are extracted and converted to a one-or-zero vector. The CNN network is trained using the 2D-coded feature vector. The low-resource model analyzes real-time apps on mobile devices. The malware detection rate (accuracy) is 96.2%, with precision, recall, and F-scores of 97.9%, 98.2%, and 98.1%, respectively. Kumar et al. [16] use an AVClass tool and a clustering technique to systematically label the binary samples. The labelled malicious program is shown in grayscale images so that local and global textural features can be extracted. The stacking of ensemble feature maps is generated from various image descriptors. The test accuracy for the suggested method is 98.34%. Ma et al. [3] presented a machine-learning-based method for detecting Android malware. The CFG features are extracted to get API information. Three Android malware detection models are constructed based on API calls, frequency, and sequence. The final step is to create a conforming ensemble model. The detection model achieves 98.98% accuracy. Frenklach et al. [17] recommended a technique for examining static Android apps based on an app similarity graph (ASG). The key to categorizing an app’s activity is found in its generic, reusable key components, such as functions, it is assumed. Using the Drebin benchmark dataset and a dataset provided by VirusTotal, the proposed work achieved an accuracy of 97.5% and an AUC score of 98.7% in balanced settings, respectively. Nguyen et al. [18] suggested a method called PSI-Graph, which analyzes function-call graphs for each executable file, to identify IoT botnets. The experimental findings show that, when applied to a dataset of 11,200 ELF files containing 7199 IoT botnet samples and 4001 benign samples, the suggested method attains an accuracy of 98.7%. Pektas et al. [19] used the API call graph to demonstrate all possible malware execution paths. Deep neural networks embed API call graphs as low-dimensional numeric vectors. This study concentrates on optimizing network performance by investigating distinct encoding algorithms and tuning network configuration parameters to ensure the best hyperparameter mixture and maximum metric value. The proposed method achieves 98.86% accuracy, 98.65% F-measure, 98.47% recall, and 98.84% precision. Our proposed method utilized the hybrid features of CFG and multi-model features for malware detection and classification. It is shown that the proposed approach outperformed as compared to the recently published works.

**Table 10:** Performance comparison of transfer learning methods with the proposed approach

Reference	Methods	Accuracy (%)
Arslan et al. [15]	CNN	96.2
Kumar et al. [16]	Stacked Ensemble	98.34
Ma et al. [3]	CFG & Ensemble	98.98
Frenklach et al. [17]	App Similarity Graph (ASG)	97.5
Nguyen et al. [18]	Function-call Graph & DCNN	98.7
Pektas et al. [19]	API-call Graph & DNN	98.65
Our Proposed	Hybrid (CFG & multi-model image)	99.27

### Computational complexity

Computational Complexity (CC), is concerned with categorizing computational issues based on their resource utilization and relating these classes to one another. We analyzed CC for each algorithm presented in Table 11,^2^. The complexity is based on the space required for the proposed approach.

**Table 11:** CC of our proposed approach

Algorithms	*APK*	*JDEX*/*DEX*	*Extractor*	*TTF*/*TF*/*Dt*/*CF*/*DF*	*CFG*/*ACG*	BL/ML
Algorithm 1	2|*n*|	$|R|+|.d|+|M|$	|*n*|	−	−	−
Algorithm 2	−	−	−	|*n*|	|*n*|	−
Algorithm 3	−	−	−	3|*n*|	−	−
Algorithm 4	−	−	−	$5|n|+|\frac{n}{4}|$	−	$6|BL|+2|ML|$

## Conclusion

Android is the most popular mobile operating system, making it an appealing pinpoint for cyber actors. Consequently, it is essential to evade these threats efficiently. Machine learning is a viable solution for malware detection, which is heavily reliant on features. Despite the numerous features of these malware analyzers, cyber actors can avoid detection by understanding the features. Consequently, one of the main duties of the Android security sector is to consistently propose cutting-edge features that can spot fraudulent behaviour. This paper describes a novel feature extraction method for detecting attacks that combines ACGs and malware images. To extract the DEX file and Java source code from an APK, reverse engineering is used. We generate an ACG to represent Android apps with elevated characteristics by harvesting API-Calls from CFGs. The ACGs can be used to generate a digital fingerprint of Android app activity. The trained features vector is then retrieved from ACGs using the attention-based transfer learning method with multiple heads. The DEX file is turned into a malware image, and texture features are extracted and outlined. Finally, the ACGs and texture features are combined to effectively detect and classify malware. The proposed method achieves the highest classification accuracy, 99.27%, when utilizing a CIC-InvesAndMal2019-customized dataset. Extensive experiments are also carried out to compare the proposed method with state-of-the-art transfer approaches, as it has been demonstrated that our methods outperform. The BERT-base with texture features is the next best method for achieving good results, with 98.52% classification accuracy. Compared to using a single type of feature, it is demonstrated that hybrid features provide outstanding classification results.

In the future, the trained features can eventually be mined using GloVe and Fast-text trained models. In addition, the effectiveness of malware detection can be assessed using more sophisticated deep learning models, such as reinforcement learning.

## Data Availability

Not applicable.

## Footnotes

1 http://api.airpush.com/v2/api.php

2 Where R stands for resources, .d for .dex, BL for base learner, and ML for machine learning.
